# Minerals and chelated-based manganese fertilization influences the productivity, uptake, and mobilization of manganese in wheat (*Triticum aestivum* L.) in sandy loam soils

**DOI:** 10.3389/fpls.2023.1163528

**Published:** 2023-06-08

**Authors:** Salwinder Singh Dhaliwal, Vivek Sharma, Arvind Kumar Shukla, Vibha Verma, Manmeet Kaur, Amnah Mohammed Alsuhaibani, Ahmed Gaber, Prabhjot Singh, Alison M. Laing, Akbar Hossain

**Affiliations:** ^1^ Department of Soil Science, Punjab Agricultural University, Ludhiana, India; ^2^ Indian Council of Agricultural Research (ICAR), Indian Institute of Soil Science, Berasia Rd, Navi Bagh, Bhopal, Madhya Pradesh, India; ^3^ Department of Chemistry, Punjab Agricultural University, Ludhiana, India; ^4^ Department of Physical Sport Science, College of Education, Princess Nourah bint Abdulrahman University, Riyadh, Saudi Arabia; ^5^ Department of Biology, College of Science, Taif University, Taif, Saudi Arabia; ^6^ Agriculture & Food, Commonwealth Scientific and Industrial Research Organisation (CSIRO), Brisbane, QLD, Australia; ^7^ Division of Soil Science, Bangladesh Wheat and Maize Research Institute, Dinajpur, Bangladesh

**Keywords:** wheat, manganese, fertilizer, yield, uptake

## Abstract

Manganese (Mn) is an essential micronutrient in plants, and it is necessary for hydrolysis in photosystem II, chlorophyll biosynthesis, and also chloroplast breakdown. Limited Mn availability in light soil resulted in interveinal chlorosis, poor root development, and the development of fewer tillers, particularly staple cereals including wheat, while foliar Mn fertilizers were found efficient in improving crop yield as well as Mn use efficiency. In the above context, a study was conducted in consecutive two wheat growing seasons for screening of the most effective and economical Mn treatment for improving the yield and Mn uptake in wheat and to compare the relative effectiveness of MnCO_3_ against the recommended dose of MnSO_4_ for wheat. To fulfill the aims of the study, three manganese products, namely, 1) manganese carbonate MnCO_3_ (26% Mn w/w and 3.3% N w/w), 2) 0.5% MnSO_4_·H_2_O (30.5% Mn), and 3) Mn-EDTA solution (12% Mn), were used as experimental treatments. Treatments and their combinations were as follows: two levels of MnCO_3_ (26% Mn) @ 750 and 1,250 ml ha^−1^ were applied at the two stages (i.e., 25–30 and 35–40 days after sowing) of wheat, and three sprays each of 0.5% MnSO_4_ (30.5% Mn) and Mn-EDTA (12% Mn) solution were applied in other plots. The 2-year study showed that Mn application significantly increased the plant height, productive tillers plant^−1^, and 1,000 grain weight irrespective of fertilizer source. The results of MnSO_4_ for grain yield wheat as well as uptake of Mn were statistically at par with both levels (750 and 1,250 ml ha^−1^) of MnCO_3_ with two sprays at two stages of wheat. However, the application of Mn in the form of 0.5% MnSO_4_·H_2_O (30.5% Mn) was found more economical than MnCO_3_, while the mobilization efficiency index (1.56) was found maximum when Mn was applied in MnCO_3_ with two sprays (750 and 1,250 ml ha^−1^) in the two stages of wheat. Thus, the present study revealed that MnCO_3_ can be used as an alternative to MnSO_4_ to enhance the yield and Mn uptake of wheat.

## 1 Introduction

Wheat (*Triticum aestivum* L.) is one of the oldest and most important cereal crops on Earth (Akhtar et al., 2018; Dhaliwal et al., 2019). It contains carbohydrates, protein, fiber, many vitamins, and many macro- and micronutrients (Igrejas and Branlard, 2020). Nearly 50% of the world’s population suffers from micronutrient deficiency such as manganese (Mn) because of lower quality of food consumption (Aziz et al., 2019). In developing countries, the health index is falling because of reduced nutrition levels in diet including Mn (Jankowska et al., 2012).

Mn is an essential micronutrient in plants since it is linked to the hydrolysis in photosystem II, chlorophyll biosynthesis, and also chloroplast breakdown. Mn unavailability in soil, particularly in light soil, causes interveinal chlorosis, poor root development, and the development of fewer tillers, particularly wheat (Lu et al., 2004; Alejandro et al., 2020, Cakmak, 2008). In plants, Mn acts as a catalyst in several enzymatic reactions and is involved in the plant’s respiratory process where it manages the redox potential of plant cells under light and dark phases (Millaleo et al., 2010). Manganese deficiency severely affects plant carbohydrates and pollen fertility during grain filling, which results in the reduction of crop yield (Marschner, 1995). Mn deficiency significantly decreases photosynthesis efficiency, reducing plant productivity and dry matter yield (Schmidt et al., 2016; Rashed et al., 2019).

Continuous use of chemical fertilizers affects agricultural sustainability, depleting the micronutrient status of soils through removal, consequently affecting plant, animal, and human health through malnutrition (Nadim et al., 2011). Many problems and disorders in plants, animals, and humans could be overcome with the intake of Mn-enriched cereals rather than consumption through additional dietary supplements. Manganese-enriched grains of wheat result in improved seedling vigor and denser stands and possess greater potential for stress tolerance.

Plants can easily absorb soluble compounds of Mn by foliar application (Katyal and Rattan, 2003). In one of the studies, the foliar application of Mn showed that nutrients supplied would be absorbed and transported from the application point (leaf foliage) to the growing tissues (Nayyar et al., 1985; Dhaliwal et al., 2021b). The foliar application of MnSO_4_ in deficient soils enhanced both wheat grain yield and concentration of Mn in grains. Different interventions are currently being used to tackle Mn deficiency in animals and humans.

In Punjab, India, the change in the pattern of crops from cotton–wheat and maize–wheat to rice–wheat has increased the iron (Fe) deficiency in rice and, thus, deficiency of Mn in succeeding wheat (Dhaliwal et al., 2011; Dhaliwal et al., 2022a). To overcome Mn deficiency, food fortification and supplementation are being extensively used in certain areas (Dhaliwal et al., 2022b). However, these approaches are very expensive and not easily accessible to developing countries (Bansal et al., 1994). Among these different strategies, fortification is considered sustainable (Dhaliwal et al., 2014) and cost-effective in enhancing Mn concentration in wheat grains. Ferti-fortification of wheat grains with micronutrients is a better way to increase micronutrient concentration in grains.

As far as different methods are concerned, the foliar spray of Mn is found to be more efficient as compared to the application in the soil of plants’ roots. One of the studies indicated that the foliar spray with Mn in wheat significantly increased the yield from 1.44% to 5.15% and Mn concentration from 24.0% to 28.25% (Dhaliwal et al., 2011). Several pieces of evidence show that foliar sprays of Mn (ferti-fortification) under field conditions are greatly efficient and the most practical method to enhance yield as well as accumulation of Mn in wheat (Nayyar et al., 1990). Less literature has been reported regarding the effect of Mn fortification on yield, content, and Mn uptake in wheat. The main hypothesis involved in this study was that the Mn application would strengthen yield, content, Mn uptake, and wheat profitability. Thus, this work focused on the screening of the most effective and economical Mn treatment for improving the yield and Mn uptake in wheat and comparing the relative effectiveness of MnCO_3_ against the recommended dose of MnSO_4_ for wheat.

## 2 Materials and methods

### 2.1 Site specification

The present experiment was conducted in a randomized block design (RBD) involving three replications, throughout two rabi (winter) seasons of 2018–2019 and 2019–2020 at Research Farm, Department of Soil Science, Punjab Agricultural University (PAU), Ludhiana (30°56′N, 75°52′E, and 247 m above mean sea level). The experimental soil was sandy loam in texture exhibiting pH, electrical conductivity (EC), and soil organic carbon values of 7.3, 0.36 dS m^−1^, and 3.1 g kg^−1^, respectively (Walkley and Black, 1934; Jackson, 1973). The original contents of micronutrients Zn, Fe, Mn, and Cu were 1.10, 5.44, 3.01, and 0.80 mg kg^−1^, respectively, in the experimental soil (Lindsay and Norvell, 1978).

### 2.2 Experimental details

The wheat variety ‘HD 3086’ used for the experiment was sown during November 2018–2019 and 2019–2020. The recommended dose of NPK (i.e., N @ 125 kg ha^−1^, P_2_O_5_ @ 62.5 kg ha^−1^, and K_2_O @ 40 kg ha^−1^) was given to all the plots. Wheat was sown by using the drill method where the spacing between the rows was 22.5 cm. To enrich the yield and uptake of wheat plants with Mn, the following three manganese products were used as experimental treatments: 1 manganese carbonate MnCO_3_ (26% Mn w/w and 3.3% N w/w), 2) 0.5% MnSO_4_·H_2_O (30.5% Mn), and 3) Mn-EDTA solution (12% Mn). Treatments and their combinations were as follows: two levels of MnCO_3_ (26% Mn) @ 750 and 1,250 ml ha^−1^ were applied at the two stages (i.e., 25–30 and 35–40 days after sowing) of wheat, and three sprays of each 0.5% MnSO_4_ (30.5% Mn) and Mn-EDTA (12% Mn) solution were applied in other plots. Manganese carbonate (26% Mn) used was YaraVita Mantrac liquid manganese fertilizers, and analytical grade fertilizers MnSO_4_·H_2_O and Mn-EDTA were applied as a spray application. Different treatment combinations in the experimental field are reported in **Table 1**.

**Table 1:** Details of the treatments used in the current study.

Short form	Treatments’ detail
T1	Application of 750 ml ha^−1^ (MnCO_3_) at 35–40 days after sowing along with recommended NPK fertilizer (i.e., N @ 125 kg ha^−1^, P_2_O_5_ @ 62.5 kg ha^−1^, and K_2_O @ 40 kg ha^−1^)
T2	Application of 1,250 ml ha^−1^ (MnCO_3_) at 35–40 days after sowing along with recommended NPK fertilizer (i.e., N @ 125 kg ha^−1^, P_2_O_5_ @ 62.5 kg ha^−1^, and K_2_O @ 40 kg ha^−1^)
T3	Application of 750 ml ha^−1^ (MnCO_3_) at 25–30 days after sowing and at 35–40 days after sowing along with recommended NPK fertilizer (i.e., N @ 125 kg ha^−1^, P_2_O_5_ @ 62.5 kg ha^−1^, and K_2_O @ 40 kg ha^−1^)
T4	Application of 1,250 ml ha^−1^ (MnCO_3_) at 25–30 days after sowing and at 35–40 days after sowing along with recommended NPK fertilizer (i.e., N @ 125 kg ha^−1^, P_2_O_5_ @ 62.5 kg ha^−1^, and K_2_O @ 40 kg ha^−1^)
T5	Application of a recommended dose of MnSO_4_·7H_2_O along with recommended NPK fertilizer (i.e., N @ 125 kg ha^−1^, P_2_O_5_ @ 62.5 kg ha^−1^, and K_2_O @ 40 kg ha^−1^)
T6	Application of a recommended dose of Mn-EDTA along with recommended NPK fertilizer (i.e., N @ 125 kg ha^−1^, P_2_O_5_ @ 62.5 kg ha^−1^, and K_2_O @ 40 kg ha^−1^)
T7	Control (only recommended NPK fertilizer: N @ 125 kg ha^−1^, P_2_O_5_ @ 62.5 kg ha^−1^, and K_2_O @ 40 kg ha^−1^ were applied to wheat)

The recommended dose of MnSO4·7H2O and Mn-EDTA: one spray of 0.5% of each solution, 2 days before first irrigation and two sprays afterward at weekly intervals. Recommended NPK fertilizer: N @ 125 kg ha−1, P2O5 @ 62.5 kg ha−1, and K2O @ 40 kg ha−1 were applied to wheat.

### 2.3 Estimation of plant growth parameters and yield

At the stage of physiological maturity, plants were harvested manually, and the grain and straw samples were taken for further study. Yield (grain and straw) was calculated from the net plot area ignoring the border rows, which were further measured in kg ha^−1^. The parameters associated with growth were calculated by randomly selecting five plant samples from central rows. The height of the plant from base to tip was noted by using a meter scale, and the mean height was expressed in cm. The total tillers at various intervals and the number of productive tillers at maturity were counted manually.

### 2.4 Estimation of Mn concentration and uptake in plant samples

The samples (grain and straw) were dried in a hot air oven for 3 days at 65°C and were ground using a Wiley mill. The concentration of Mn was calculated through the wet-acid digestion method, where the samples were digested with a mixture of di-acids (HNO_3_ and HClO_4_ in a ratio of 4:1) (Piper, 1966). After digestion, the samples were analyzed for the total content of micronutrients through atomic absorption spectrophotometry (AAS) (Page et al., 1982).

The uptake of Mn was computed from Eq. 1:


(Eq.1) $$\text{Uptake }\left({\text{g ha}}^{-1}\right)\;=\frac{\text{Mn content }\left({\text{mg kg}}^{-1}\right)\;\times \text{ Yield }\left({\text{kg ha}}^{-1}\right)}{1,000}.$$

### 2.5 Estimation of harvest index, physiological efficiency, and mobilization efficiency index

The harvest index (HI), physiological efficiency (PE), and mobilization efficiency index (MEI) were calculated using the formulas reported in the literature (Dhaliwal et al., 2021a). The harvest index refers to the ratio of grain yield to biological yield and was computed through Eq. 2:


(Eq. 2) $$\text{Harvest index }=\frac{\text{Grain yield }\left({\text{kg ha}}^{-1}\right)}{\text{Grain yield }\left({\text{kg ha}}^{-1}\right)\;+\text{ straw yield }\left({\text{kg ha}}^{-1}\right)}.$$

Physiological efficiency was calculated using Eq. 3.


(Eq. 3) $$\text{PE}=\frac{{\text{Y}}_{\text{t}}-{\text{Y}}_{\text{c}}}{{\text{NU}}_{\text{t}}-{\text{NU}}_{\text{c}}},$$

Y_t_ and Y_c_ are the grain yield (kg ha^−1^) of wheat in plots fertilized with Mn and control, respectively; NU_t_ and NU_c_ are the Mn uptake (g ha^−1^) of wheat in plots fertilized with Mn and control, respectively.

The mobilization efficiency index was computed by using Eq. 4:


(4) $$\text{MEI}=\frac{\text{Mn concentration in grain}}{\text{Mn concentration in straw }}.$$

### 2.6 Economic analysis

The independent calculation for the cost of fertilizer pertaining to different treatments was in United States dollars (USD) per hectare while considering the fertilizer cost at the application time. The calculation of gross return was from the minimum support price (MSP) of wheat set by the government of India through the formula given in Eq. 5. This was further used to calculate net return (Eq. 6) and B:C (benefit:cost) ratio (Eq. 7) in the present study.


Eq. (5) Gross return=Yield ×Price of produce,

Eq. (6) $$\text{Net return }\left({\text{USD ha}}^{-1}\right)=\left(\text{Gross return}-\text{Cost of cultivation}\right)\left({\text{USD ha}}^{-1}\right),$$

Eq. (7) $$\text{B}:\text{C ratio}=\;\frac{\text{Gross return }}{\text{Cost of cultivation}}.$$

### 2.7 Statistical analysis

Statistical analysis of the results was performed by using the SPSS package, version 16.0 (SPSS Inc., Chicago, IL, USA). Different means and differences between the values of all studied parameters were compared using a one-way analysis of variance through Duncan’s multiple range test (DMRT), which included a probability level of 0.05.

## 3 Results

### 3.1 Minerals and chelated-based Mn fertilization effect on plant growth parameters

Different plant parameters, viz., plant height at maximum tillering (cm), plant height at maturity (cm), tillers plant^−1^ at maximum tillering stage, productive tillers plant^−1^ at maturity, and 1,000 grain weight were reported in the experimental field at various stages. Plant height at maximum tillering showed non-significant results with the highest value under treatment T4 (41.8 cm) involving the foliar application of MnCO_3_ (26% Mn) fertilizer @ 1,250 ml ha^−1^ at 25–30 and 35–40 days after sowing followed by T6 (41.7 cm) involving application of Mn-EDTA along with recommended NPK fertilizer, whereas treatment T7, i.e., control, exhibited minimum plant height of 37.7 cm (**Table 2**).

**Table 2:** Effect of different sources of Mn fertilizers at different stages on different plant parameters of wheat.

Treatments	Plant height at max. tillering stage (cm)	Plant height at maturity (cm)	Tillers plant^−1^ at max. tillering stage (no.)	Productive tillers plant^−1^ at maturity (no.)	1,000 grain weight (gm)
T1	39.9	100.1^a^	114.7	87.7^a^	38.6^bc^
T2	40.2	100.0^a^	116.0	87.8^a^	40.2^ab^
T3	41.4	101.5^a^	117.0	85.8^a^	39.9^abc^
T4	41.8	103.2^a^	118.2	88.0^a^	42.8^a^
T5	41.2	102.7^a^	119.0	88.2^a^	42.8^a^
T6	41.7	101.6^a^	118.3	88.3^a^	42.6^a^
T7	37.7	95.3^b^	113.0	73.7^b^	36.7^c^
LSD _0.05_	NS	4.6	NS	7.8	3.2
CV (%)	11.6	7.8	9.4	8.5	6.7

Treatment details are given in **Table 1**. Values with similar letters in superscript show that they do not differ significantly at 5% level according to Duncan’s multiple range test. LSD, Least Significant Difference and CV, Coefficient of Variation.

On the one hand, plant height at maturity was maximum in treatment T4 (103.2 cm) and was not statistically different from the rest of the treatments excluding treatment T7. On the other hand, tillers/plant at maximum tillering and maturity stage was again the highest under treatment T6 with values of 118.3 and 88.3, respectively. Additionally, treatments involving foliar application of MnCO_3_ (26% Mn) fertilizer @ 1,250 ml ha^−1^ at 25–30 and 35–40 days after sowing (T4) and MnSO_4_ (T5) were the most efficient in improving 1,000 grain weight with the highest value of 42.8 g, where both treatments were not statistically different from each other (**Table 2**).

### 3.2 Minerals and chelated-based Mn fertilization effect on the productivity of wheat

Mean data of a 2-year study reported a significant improvement in wheat yield through the use of manganese sulfate (MnSO_4_) and manganese carbonate (MnCO_3_) fertilizer over no fertilizer application as presented in **Table 3**.

**Table 3:** Effect of different sources of Mn fertilizers at different stages on the yield of wheat.

Treatments	Grain yield (kg ha^−1^)			Straw yield (kg ha^−1^)		
	2018–2019	2019–2020	Average	2018–2019	2019–2020	Average
T1	4,967^c^	5,140^c^	5,054^c^	9,326^ab^	8,937^bc^	9,131^bc^
T2	4,999^c^	5,240^bc^	5,119^bc^	9,025^b^	8,860^bc^	8,942^c^
T3	5,439^ab^	5,553^ab^	5,496^a^	8,987^b^	9,236^abc^	9,111^bc^
T4	5,596^a^	5,597^a^	5,596^a^	9,462^ab^	9,307^ab^	9,384^ab^
T5	5,579^a^	5,627^a^	5,603^a^	9,717^a^	9,662^a^	9,689^a^
T6	5,165^bc^	5,233^c^	5,199^b^	9,266^ab^	8,850^c^	9,058^bc^
T7	4,618^d^	4,703^d^	4,661^d^	7,621^c^	7,417^d^	7,519^d^
LSD _0.05_	323	315	143	612	450	393
CV (%)	8.3	7.7	6.8	9.9	8.2	6.4

Treatment details are given in **Table 1**. Values with similar letters in superscript show that they do not differ significantly at 5% level according to Duncan’s multiple range test. LSD, Least Significant Difference and CV, Coefficient of Variation.

Treatment T5 showed the highest grain (5,603 kg ha^−1^) and straw (9,689 kg ha^−1^) yield of wheat where MnSO_4_ along with recommended NPK fertilizers was applied followed by grain (5,596 kg ha^−1^) and straw (9,384 kg ha^−1^) yield through the foliar application of MnCO_3_ (26% Mn) fertilizer @ 1,250 ml ha^−1^ at 25–30 and 35–40 days after sowing (treatment T4). Both treatments were not statistically different from each other for grain and straw yield. Additionally, the foliar application of Mn-EDTA showed a decreased yield of 5,199 and 9,058 kg ha^−1^ for grain and straw, respectively, in comparison to the sole application of MnSO_4_. Also, the single dose of MnCO_3_ fertilizer @ 750 (T1) and 1,250 ml ha^−1^ (T2) at 25–30 days after sowing resulted in lesser grain (5,054 and 5,119 kg ha^−1^) as well as straw yield (9,131 and 8,942 kg ha^−1^) in comparison to the double dose of MnCO_3_ fertilizer (T3 and T4). However, treatment T7 (control) showed minimum grain and straw yield of 4,661 and 7,519 kg ha^−1^, respectively.

### 3.3 Minerals and chelated-based Mn fertilization effect on grain and straw Mn concentration

**Figure 1:**
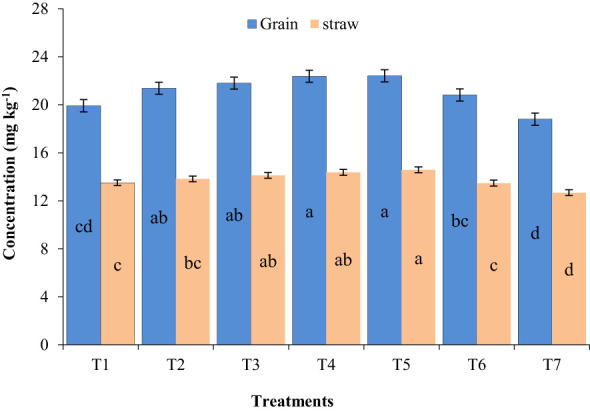
Effect of different sources of Mn fertilizers at different stages on Mn concentration of wheat. Treatment details are given in **Table 1**. Values with similar letters show that they do not differ significantly at 5% level of probability as per Duncan’s multiple range test.

The average data for Mn concentration in wheat are presented in **Figure 1**. Manganese concentration of wheat enhanced significantly with the foliar application of Mn as compared to control with values varying at 18.80–22.41 and 12.68–14.58 mg kg^−1^ for grain and straw, respectively.

Treatment T5 comprising MnSO_4_ foliar application along with the recommended NPK fertilizer exhibited the highest Mn content in both grain (22.41 mg kg^−1^) and straw (14.58 mg kg^−1^) of wheat, followed by T4 (22.38 and 14.37 mg kg^−1^, respectively) involving foliar use of MnCO_3_ (26% Mn) fertilizer @ 1,250 ml ha^−1^ at 25–30 and 35–40 days after sowing, which were not statistically different from each other. Additionally, Mn-EDTA application in treatment T6 resulted in decreased Mn content in both grain and straw of wheat in comparison to the treatments with MnCO_3_ and MnSO_4_ alone.

### 3.4 Minerals and chelated-based Mn fertilization effect on Mn uptake in grain and straw of wheat

A significant increase in Mn grain and straw uptake was observed with Mn application through different fertilizer sources in comparison to the control treatment (**Table 4**). The average data revealed that the highest value of Mn uptake by grain (43.36%) and straw (48.14%) was recorded in treatment T5. The Mn uptake by grain in treatment T5 was not statistically different from that in treatments T3 and T4. Likewise, the straw Mn uptake in treatment T5 was statistically at par with that in treatment T4.

**Table 4:** Effect of different sources of Mn fertilizers on its grain and straw uptake in wheat.

Treatments	Mn uptakes (g ha^−1^)					
	Grain			Straw		
	2018–2019	2019–2020	Average	2018–2019	2019–2020	Average
T1	99.38^c^	101.94^d^	100.66^c^	128.23^bc^	118.44^c^	123.34^c^
T2	106.73^bc^	112.22^bc^	109.48^b^	125.59^c^	121.68^bc^	123.64^c^
T3	120.57^a^	119.12^ab^	119.84^a^	126.71^c^	130.53^ab^	128.62^bc^
T4	125.16^a^	125.66^a^	125.41^a^	136.72^ab^	133.02^a^	134.87^ab^
T5	127.26^a^	123.59^a^	125.43^a^	145.18^a^	137.32^a^	141.26^a^
T6	112.09^b^	104.32^cd^	108.20^bc^	128.65^bc^	115.64^c^	122.14^c^
T7	90.21^d^	84.97^e^	87.59^d^	97.99^d^	92.71^d^	95.35^d^
LSD _0.05_	7.98	8.25	8.12	9.54	9.13	9.47
CV (%)	7.8	6.1	6.6	9.5	8.2	10.7

Treatment details are given in **Table 1**. Values with similar letters in a superscript show that they do not differ significantly at 5% level of probability according to Duncan’s multiple range test. LSD, Least Significant Difference and CV, Coefficient of Variation.

### 3.5 Minerals and chelated-based Mn fertilization effect on Mn use efficiencies and harvest index

The HI value was maximum in treatment T7 (38.27) and minimum in treatment T1 (35.63). The HI increased with the increase in the supply of MnCO_3_ (**Table 5**). The results also stated that the HI of T5 and T6 was higher than that of T1 and T2 but lower than that of T3 and T4. The maximum value of PE was found in treatment T1 (48.78), whereas the minimum value was observed in treatment T4 (36.28). Similarly, the MEI was maximum in treatment T4 (1.56) and minimum in treatments T1 and T7 (1.48). The MEI also increased with the increase in Mn supply except for T1.

**Table 5:** Effect of different sources of Mn fertilizers on harvest index, physiological efficiency, and mobilization efficiency index of wheat.

Treatments	HI (%)	PE	MEI
T1	35.63	48.78	1.48
T2	36.41	37.48	1.55
T3	37.63	37.02	1.54
T4	37.38	36.28	1.56
T5	36.61	37.07	1.54
T6	36.47	43.79	1.54
T7	38.27	–	1.48

Treatment details are available in **Table 1**.

HI, harvest index; PE, physiological efficiency; MEI, mobilization efficiency index.

### 3.6 Economic analysis for minerals and chelated-based Mn fertilization

**Figure 2:**
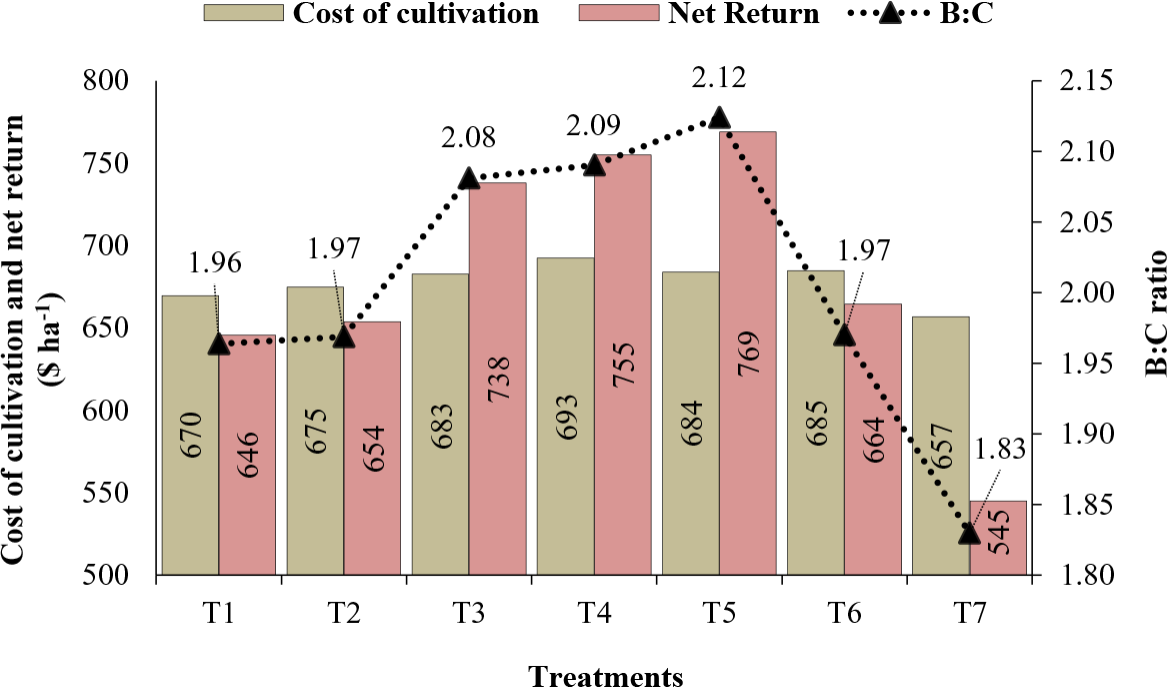
Economics of wheat cultivation as affected by different Mn fertilizer sources.

The effect of MnCO_3_, MnSO_4_, and Mn-EDTA foliar application at different phases on wheat economics is given in **Figure 2**.

The maximum cost of cultivation was found in treatment T4 ($693) and T6 ($685), whereas treatment T7 ($657) showed the minimum cost. Net return was found the highest in T5 ($769) followed by T4 ($755). Also, the benefit:cost (B:C) ratio displayed a higher value in T5 (2.12) and lower in T7 (1.83). The cost of cultivation, net return, and B:C ratio were positively affected by the treatment of Mn.

## 4 Discussion

### 4.1 Minerals and chelated-based Mn influence the growth of wheat

Plant height is an important parameter involved in plant development, which helps in predicting the growth rate and yield. Plant height at the maximum tillering stage showed non-significant variation, whereas plant height at maturity significantly increased with the foliar application of Mn because of its structural role in chlorophyll. The Mn was applied through foliar spray over plants, which resulted in increased chlorophyll content of the leaves and improved plant height. The tillers/plant is a factor that affects grain yield because it not only indicates how well a crop is established but also results in a greater number of grains, which raises the yield of wheat crops. Because of improved pollen germination and fertilization, Mn application increased the productive tillers and seed set (Goussias et al., 2002). Manal et al. (2010) reported an increased number of tillers through the Mn application. However, an increase in 1,000 grain weight through Mn application might be linked to the enhanced relationship between source and sink, which ensured the maximum supply of assimilates at the time of grain development (Longnecker et al., 1991; El-Esawi and Sammour, 2014; El-Esawi et al., 2015).

### 4.2 Minerals and chelated-based Mn influence the productivity of wheat

Enhanced yields due to MnSO_4_ and MnCO_3_ application along with a recommended dose of NPK might be because of improved soil characteristics and crop productivity. In general, Mn deficiency leads to improper development of anther, infertility in pollen, and reduced supply of assimilates, which lead to reduced grain setting and yield (Nadeem and Farooq, 2019). Manganese foliar application to wheat at various stages efficiently translocated Mn toward the parts involved in reproduction and then accumulated in grains (Li et al., 2014; Zulfiqar et al., 2020). Manganese foliar application also caused absorption in the leaf epidermis, and following re-mobilization, it was delivered to growing grains through the xylem, thus increasing grain yield (Mousavi et al., 2007). Additionally, Mn functions as a co-factor for the Mn^2+^-dependent superoxide dismutase enzyme and several tricarboxylic cycle enzymes in the pathway of shikimic acid that produces aromatic amino acids (Marschner, 1995). Apart from its biological function in plants, Mn is essential for the photosynthetic process because it catalyzes the breakdown of water molecules taking place under light during photosystem II (PSII) and the RuBP carboxylase reaction, which increases the yield of grain and straw in wheat (Marschner et al., 1986, Marschner, 1995). Without Mn, photosynthesis cannot be performed, as it is a dominant part of the complex evolving oxygen during photosystem II (Malavolta et al., 1997). A sufficient level of Mn enhanced the grain and straw yield through better fertilization, grain setting, and assimilate supply. Additionally, the Mn-EDTA application showed decreased grain and straw yields in comparison to the sole application of MnSO_4_ in this study. This might be due to the formation of Mn and EDTA complex where the release of Mn and its participation in metabolic processes was restricted. This resulted in lesser Mn availability to crops, which further decreased the wheat yield.

### 4.3 Minerals and chelated-based Mn influence Mn concentration in grain and straw

The foliar application of MnCO_3_ and MnSO_4_ showed a significant increase in Mn concentration over control because of the quick absorption of Mn through plant leaves (**Figure 1**). Higher absorption levels as well as assimilation of Mn added nutritional value to the crop, resulting in increased growth and nutrients required for improving food quality. In the present experiment, foliar-applied Mn improved the grain Mn content as compared to straw, which might be because of accumulated Mn on flag leaf, which was better translocated toward grain, offering an adequate level of photosynthates among vegetative and reproductive parts (Zulfiqar et al., 2021). However, treatment involving Mn-EDTA application led to a lower increase in Mn concentration in comparison to other treatments. In general, EDTA is not responsible for any physiological changes and does not act as a transporter in plants (Nowack et al., 2008).

### 4.4 Minerals and chelated-based Mn influence Mn uptake by grain and straw

The significant increase in Mn uptake with Mn application was because of the greater Mn absorption in the wheat foliage. Also, an increased Mn uptake in grain due to the enhanced rates of MnCO_3_ and MnSO_4_ application was observed (Kanubhai, 2013). Manganese in its reduced state, i.e., Mn^2+^, acts as the only available form to plants that could be taken up by epidermal root cells through an active transport system and could be further translocated as the divalent cation into the plant (Gherardi and Rengel, 2003; Pittman, 2005). Additionally, the uptake of Mn in straw samples of wheat was significantly different where the Mn uptake by roots occurs through xylem tissue in the plant. Further, a higher correlation of Mn influx with its uptake resulted in a direct supply of Mn to the straw, hence leading to the increased Mn uptake in wheat straw (Jhanji et al., 2014). Microbial as well as chemical mobilization also helps in increasing the Mn solubility, which enhances the uptake of Mn in wheat (Abbas et al., 2009).

### 4.5 Minerals and chelated-based Mn influence the Mn use efficiencies and harvest index

The data associated with HI showed that HI increased with the increase in MnCO_3_ supply, which showed that MnCO_3_ increased grain production over the total biological yield. The highest HI in the control treatment showed that the overall grain-to-straw ratio was the highest in the untreated plot over the treated plots. Thus, Mn supply increased the total dry matter production over grain yield alone, which is linked to the HI of wheat and its use efficiency. The PE refers to the plant’s ability to transform Mn acquired from fertilizer into economic yield. As the PE was the highest in treatment T1, it is suggested that the plant with a lower Mn supply has a higher tendency to transform it into yield, and afterward, excess Mn supply causes an increase in Mn concentration in plants. The MEI depicts the mobilization ratio of nutrients in grain to straw. The increase in MEI with the increase in Mn supply showed more Mn mobilization in grain as compared to straw, which also increases its concentration in grain. The results of the current observation on Mn use efficiency and HI were also confirmed by several earlier studies that stated that as compared to traditional micronutrient fertilization, chelated-based fertilization is significantly more effective and efficient than non-chelated source micronutrient fertilizer (Rashed et al., 2019; Alejandro et al., 2020). Sadana et al. (2005) stated that chelated-based nutrients facilitate nutrient uptake use efficiency for foliar application; it is due to the leaves of plants being coated with wax, which naturally repels water and charged substances, such as Mn. Another study stated that chelated micronutrient uptake use efficiency is higher because chelated-based micronutrients can penetrate the wax layer, thus increasing uptake use efficiency and leading to higher HI in plants (Liu et al., 2012).

### 4.6 Economic point of view of different sources of Mn

In the current study, the use of MnCO_3_, MnSO_4_, and Mn-EDTA significantly improved the economic outcomes of wheat, which was in agreement with the outcomes reported by Zulfiqar et al. (2021), where Mn treatment showed an improved B:C ratio in wheat. Also, the sole application of MnSO_4_·7H_2_O (1.0%) exhibited higher net return and B:C ratio, which shows its effectiveness as compared to Mn-EDTA as well as control. Sadana et al. (2005) and Liu et al. (2012) stated that chelated-based fertilization is an environmentally friendly and cost-effective fertilization approach than the traditional approach; it is due to the uptake and use efficiency of chelated-based fertilization, particularly micronutrient management.

## 5 Conclusion

The results of the current study revealed that the use of both MnSO_4_ and MnCO_3_ significantly improved the growth, yield, and Mn uptake in wheat over the control irrespective of the sources used for Mn application. Among these three sources of Mn, the MnCO_3_ @ 750 ml ha^−1^ and 1,250 ml ha^−1^ at 25–30 and 35–40 days after sowing of wheat may be considered an agronomically efficient option for improving growth and yield along with Mn uptake in wheat over the recommended dose of MnSO_4_. Thus, the present study revealed that MnCO_3_ can be used as an alternative to MnSO_4_ to enhance the yield and Mn uptake of wheat.

## Data availability statement

The original contributions presented in the study are included in the article/supplementary material. Further inquiries can be directed to the corresponding authors.

## Author contributions

Conceptualization: SD, VS, AS, VV, MK, and PS. Data curation: SD, AA, AG, AL, and AH. Formal analysis: SD, AA, AG, AL, and AH. Investigation: SD, VS, AS, VV, MK, and PS. Methodology: SD, VS, AS, VV, MK, and PS. Resources: SD, VS, AS, VV, MK, and PS. Software: SD, AA, AG, AL, and AH. Supervision: SD and AS. Validation: SD, VS, AS, VV, MK, and PS. Writing—original draft: SD, VS, AS, VV, MK, and PS. Writing—review and editing: SD, AA, AG, AL, and AH. Funding acquisition: SD, AA, AG, AS, and AH. All authors contributed to the article and approved the submitted version.
